# Editorial: Peptidyl-Prolyl Isomerases in Human Pathologies

**DOI:** 10.3389/fphar.2019.00794

**Published:** 2019-07-11

**Authors:** Tiziano Tuccinardi, Flavio Rizzolio

**Affiliations:** ^1^Department of Pharmacy, University of Pisa, Pisa, Italy; ^2^Department of Translational Research, Pathology Unit, National Cancer Institute-CRO-IRCSS, Aviano, Italy; ^3^Department of Molecular Sciences and Nanosystems, Ca’ Foscari University of Venezia, Venice, Italy

**Keywords:** peptidyl-prolyl isomerases, therapy, signal transduction, human pathologies, cancer

Peptidyl-prolyl isomerases (PPIases) are a group of evolutionarily conserved ubiquitous proteins expressed in both prokaryotic and eukaryotic cells. PPIases function as accelerating agents, speeding up the cis/trans conformational switch of specific substrates (Schiene-Fischer et al., 2011). Based on their affinity to immunosuppressive drugs such as cyclosporin A (CsA) and tacrolimus (FK506), they are classified into three different structurally and functionally classes: the CsA-binding cyclophilins, the FK506-binding proteins (FKBPs), and the parvulin-like PPIases, which do not bind immunosuppressants. PPIase-catalyzed isomerization can serve as a signal to other key proteins in different cellular processes such as the folding of newly synthesized proteins (Schmidpeter and Schmid, 2015), immune response (Nath and Isakov, 2015), neuronal differentiation (Ernst et al., 2018), and cell cycle control (Yeh and Means, 2007; Rizzolio et al., 2012, Rizzolio et al., 2013; Cheng et al., 2017). Furthermore, several studies reported the involvement of PPIases in virion and parasite infection (Ünal and Steinert, 2014; Ernst et al., 2018; Ünal et al., 2018), vascular diseases (Perrucci et al., 2015), neurodegeneration (Blair et al., 2015), and many types of cancer (Girardini et al., 2011; Lee et al., 2011; La Montagna et al., 2012; Lucchetti et al., 2013). On this background, PPIases have been explored as potential diagnostics (Bao et al., 2004) and therapeutic targets (Rostam et al., 2015; Wei et al., 2015; Campaner et al., 2017; Russo Spena et al., 2018, Russo Spena et al., 2019).

This research topic conveyed together experts in the field of PPIases to generate a discussion regarding the mechanism of pathogenesis, the current status, and the innovative therapeutic strategies particularly in the oncology field.

In the review of Zannini et al. entitled “Oncogenic hijacking of the PIN1 signaling network,” it was analyzed how cancer cells take over physiological signals through PIN1 at the molecular and cellular levels. The main molecular pathways such as RAS-MAPK, pRB, p53, NOTCH, c-MYC, WNT/β-CATENIN, NF-kappaB, and PI3K/AKT were critically analyzed. The role of PIN1 in cell proliferation, metabolism, and stem cell reprogramming was discussed. Overall, this manuscript elegantly discussed the different and often opposite cell fates dictated by PIN1, placing its function in a context-dependent determination.

In “PIN1 in cell cycle control and cancer,” Cheng and Tse reported how PIN1 fine tunes the cell cycle protein machinery, including the retinoblastoma protein, cyclin D1, cyclin E, p27, Cdc25C, and Wee1. All the principal steps of cell cycle progression in which PIN1 is involved were described, suggesting that PIN1 overexpression leads to cell cycle deregulation and malignant cell transformation. An analysis of the recently developed new PIN1 inhibitors was provided discussing their potential use for the treatment of different types of cancer.

El Boustani et al. offered “A guide to PIN1 function and mutations across cancers.” Through an analysis of COSMIC and cBioportal associated databases, they summarized all the mutations discovered among different types of cancer on PIN1. A molecular modeling analysis was provided to suggest the most probable activity of each single mutation and an updated summary of the most recently published inhibitors of PIN1 was also discussed.

In the research article entitled “Prolyl isomerase Pin1 directly regulates calcium/calmodulin-dependent protein kinase II activity in mouse brains” by Shimizu et al., it was speculated that PIN1 could regulate many brain diseases through calcium/calmodulin-dependent protein kinase II (CaMKII) activity. PIN1 binds CaMKII in the mouse brain and suppresses its activity. CaMKII controls many proteins involved in neuronal functional disorders, including tau, synapsin I, α-amino-3-hydroxy-5-methyl-4-isoxazolepropionic acid receptor, and N-methyl-D-aspartate receptor. Although the mechanism of action has not been elucidated yet, these data suggest new PIN1 functions in preventing neurodegeneration.

The FKBP family was described in-depth by Kolos et al. in “FKBP ligands—where we are and where to go?” In this review, the principal and well-annotated FKBP proteins in the literature were analyzed, giving a critical overview of the related pathologies. The most prominent ligands were highlighted as possible chemical tools. An interesting “keep in mind” report was provided.

Ernst et al., in their research article entitled “Combined pharmacological inhibition of cyclophilins, FK506-binding proteins, Hsp90, and Hsp70 protects cells from *Clostridium botulinum* C2 toxin,” described how the chaperone Hsp90, cyclophilin Cyp40, and FKBP51 interact with the C2I enzymatic active component and regulate its translocation into the cytosol leading to the depolymerization of F-actin. The pharmacological inhibition of Hsp90, Hsp70, Cyps, and FKBPs enhanced the protection of cells against C2 cytotoxic effects.

Unal et al., in their research paper entitled “Pleiotropic *Clostridioides difficile* cyclophilin PpiB controls cysteine-tolerance, toxin production, the central metabolism and multiple stress responses,” reported an interactomic analysis of the sole cyclophilin-type PPIase of *Clostridioides difficile* (CdPpiB). In this paper, it was found that CdPpiB interacts with the major players of transcription, translation, protein folding, stress response, and the central metabolism regulating bacterial pathogenicity.

## Author Contributions

All authors listed have made substantial, direct, and intellectual contribution to the work and approved it for publication.

## Conflict of Interest Statement

The authors declare that the research was conducted in the absence of any commercial or financial relationships that could be construed as a potential conflict of interest.
